# Usability and usefulness of U. S. federal and state public health data dashboards: implications for improving data access and use

**DOI:** 10.3389/fpubh.2025.1699312

**Published:** 2025-11-27

**Authors:** Itzhak Yanovitzky, Gretchen Stahlman, Miriam Kim

**Affiliations:** 1 School of Communication & Information, Rutgers University New Brunswick, New Brunswick, NJ, United States; 2 Florida State University School of Information, Tallahassee, FL, United States; 3 School of Information and Library Science, The University of North Carolina at Chapel Hill, Chapel Hill, NC, United States

**Keywords:** data dashboards, public health, actionability, usability, usefulness

## Abstract

**Introduction:**

Dashboards that afford timely access to credible, relevant, and actionable data can significantly improve public health decision-making at all levels. As dashboards becomes more ubiquitous, it is imperative to proactively consider how they may be optimally designed to be usable and useful to users.

**Methods:**

A cluster probability sample of U. S. federal and state public health dashboards (*N* = 210) was utilized to describe and compare common design elements and data characteristics of dashboards. A standardized valid and reliable instrument was used to extract data for assessing dashboards’ usability and usefulness.

**Results:**

Dashboards are primarily designed for epidemiological surveillance and assessing disparities. Both federal and state dashboards rely heavily on data collected by federal agencies but many state dashboards also draw on local data. Vulnerable subpopulations are underrepresented in data used in dashboards. Federal dashboards score higher than state dashboards on usability but are comparable in usefulness. About one-third of state dashboards are hosted on third-party platforms and are prone to access disruptions.

**Conclusion:**

Usability and usefulness of public health dashboards can be significantly enhanced by streamlining and enhancing users’ experience and incorporating additional customization and analytical affordances. A uniform set of best-practices and standards for optimizing dashboard design and implementation does not yet exist as research on this topic is lagging. Policy implications: Additional federal and state investments are needed to build and maintain a robust infrastructure for developing, improving, and sustaining public health dashboards and incentivize rigorous, theory grounded research to optimize usability and usefulness of dashboards.

## 1 Introduction

Timely access to credible, relevant, and actionable data is essential to making evidence-informed decisions in public health (1, 2). This requires a robust public health data infrastructure that supports critical public health functions such as epidemiological surveillance, coordinated response to health risks, designing policies that improve population health, building a skilled public health workforce, and communicating health information effectively to diverse audiences (3, 4). In this context, data dashboards are increasingly touted as a cost-effective means of supporting evidence-informed public health decision-making (5, 6). In theory, dashboards can afford timely, convenient, and near-universal access to public health data, transform complex data into intuitive information displays, and allow users the flexibility of exploring data on their own to answer questions of relevance to them (7, 8). They are also recognized by some for their evidence democratizing potential, namely supporting a process of widening access to the creation, use, and interpretation of research and data to include diverse and underrepresented stakeholders (7, 9, 10).

The current landscape of public health data dashboards is rapidly expanding to a broad range of domains, applications, and stakeholders (11–14), prompting interest in the potential utility and public health impact of these tools (5, 6, 8). As use of dashboards in public health decision-making becomes more ubiquitous, it is imperative to map and analyze the current landscape of public health dashboards and to proactively consider how they may be optimally designed to be usable and useful to a range of users (8, 15). The available research on this topic is limited, drawing primarily on findings of selective case studies of dashboards designed by researchers in academic settings, which therefore inadequately represent the significantly larger population of dashboards created and deployed by national, state, and local public health agencies (16, 17). Local and state public health agencies may not have the same access to resources and expertise needed to create, deploy, and sustain dashboards as do federal agencies and may also diverge in terms of public health issues and types of data prioritized in dashboards. In addition, systematic and rigorous assessments of usability and usefulness of public health dashboards are scarce, lacking a standard and theory-grounded conceptualization of usability and usefulness as well as empirically-tested valid and reliable measures of both constructs (17, 18). To begin addressing this gap, this study systematically mapped and analyzed the current landscape of public health data dashboards in the U. S. using a probability sample of dashboards created by federal and state health agencies. Responding to repeated calls in the field (16, 19, 20), it was intentionally designed to assess and compare usability and usefulness of federal and state dashboards using theory-grounded, valid, and reliable measures that were tested and refined iteratively to improve reproducibility. The purpose of the analysis is to identify critical gaps in current practice and propose modifications and enhancements that advance and normalize use of dashboards as means for public health decision makers at all levels to acquire and use evidence-based, actionable knowledge.

## 2 Method

### 2.1 Population and sampling

A ‘public health data dashboard’ was operationalized as a publicly accessible, active, and interactive web-based data visualization tool for presenting and analyzing public health data. This definition includes dashboards designed for presenting population-level health data (e.g., vital statistics, epidemiological and risk surveillance, environmental hazards, access or utilization of health services, health disparities, health policy, and health-related public opinion data), but excludes dashboards used in clinical settings and patient care.

Previous studies used a range of strategies to sample public health dashboards, including Google searches (21), purposive sampling (22), expert nomination (15), or combinations of these strategies (23). Since random sampling of dashboards is not feasible (24), the alternative is conducting targeted web searches for dashboards using a two-step cluster sampling strategy that treats top-level web domains used by official federal and state public health agencies (e.g.,‘cdc.gov’) as homogenous clusters and then randomly sampling a fixed number of data dashboards within each cluster (25). Applying this strategy, we compiled a list of top-level web domains used by federal public health agencies and health departments of all 50 states and five territories. We then conducted a manual web search and screening of URLs of dashboards in each top-level domain (e.g., “site: cancer.gov AND dashboard*”). This yielded a sampling framework of active federal (*n* = 358) and state (*n* = 2,158) dashboards retrieved in July 2024. Next, we employed a previously validated two-stage cluster sampling strategy to draw a probability sample of dashboards (26). This procedure involved first randomly selecting 30 clusters (top-level web domains) and then randomly selecting seven dashboards within each cluster to generate a probability sample of 210 federal and state public health dashboards (see Supplementary material for the list). All sampled dashboards were initially accessed in July 2024, reaccessed in March 2025, and again in July 2025 to verify that they are still publicly accessible and active given concerns raised in the research literature regarding the potential adverse impact of limited resources and funding on dashboards’ sustainability (6, 7, 17).

### 2.2 Data extraction

The dashboard coding instrument was developed, tested, and refined iteratively based on variables and measures adopted from similar studies that analyzed public health data dashboards (15, 16, 20, 27) and a recent scoping review of dashboard usability and usefulness measures (17). The instrument was reviewed by members of the study’s advisory group, all leading experts in design and use of public health data dashboards, and further refinements were made based on feedback they provided to improve validity. Next, five coders trained on the application of the coding instrument independently coded a random subsample of 20 dashboards to assess degree of agreement across all coding decisions (*N* = 260), and intercoder reliability was assessed using Krippendorff’s alpha which corrects for chance agreement (28). The calculated agreement coefficient (*α* = 0.87) exceeded the threshold of acceptable reliability (*α* = 0.75). Each coder was then assigned a random subsample of dashboards to code independently and a quality control procedure was implemented by senior members of the research team to detect and correct any potential errors. The coding instrument was designed to extract information regarding observable characteristics of dashboards (e.g., topics, sources and types of data used, and populations represented in the data). Usability and usefulness affordances were assessed heuristically by having coders assess adherence to recommended usability and usefulness principles (29, 30). Usability affordances coded included accessibility, ease of navigation, interactivity, data visualization, and availability of technical assistance. Key usefulness affordances assessed include (a) credibility-related information or cues (e.g., trust certificates); (b) explicit identification of target audience or intended users; (c) data customization affordances (e.g., data disaggregation or layering options); (d) data affordances (i.e., types of different questions that can be answered by the data); (e) analytical affordances (range and complexity of data analyses users can conduct); and (f) translational or interpretation affordances (features such as data storytelling that can facilitate user comprehension or ability to generate useful insights) (13, 19). A copy of the coding instrument is available from the Supplementary material.

### 2.3 Statistical analyses

Given the study’s aims, data analyses focused on describing and comparing key characteristics of national and state public health dashboards to assess similarities and differences in use, usability, and usefulness of these tools that may be due to variations in resources available to design, implement, and sustain dashboards as well as variations between national and local public health priorities. Variables were converted to multiple response items prior to generating descriptive statistics. Categories of multiple response items are not mutually exclusive and therefore violate the assumptions of standard statistical tests of differences between federal and state dashboards. For this reason, each response option on these variables was first recoded into a dummy variable, and a Pearson’s chi-square test was used to assess the statistical significance of differences between federal and state dashboards on that variable. All statistical analyses were performed using IBM SPSS version 29.

## 3 Results

**Figure 1:**
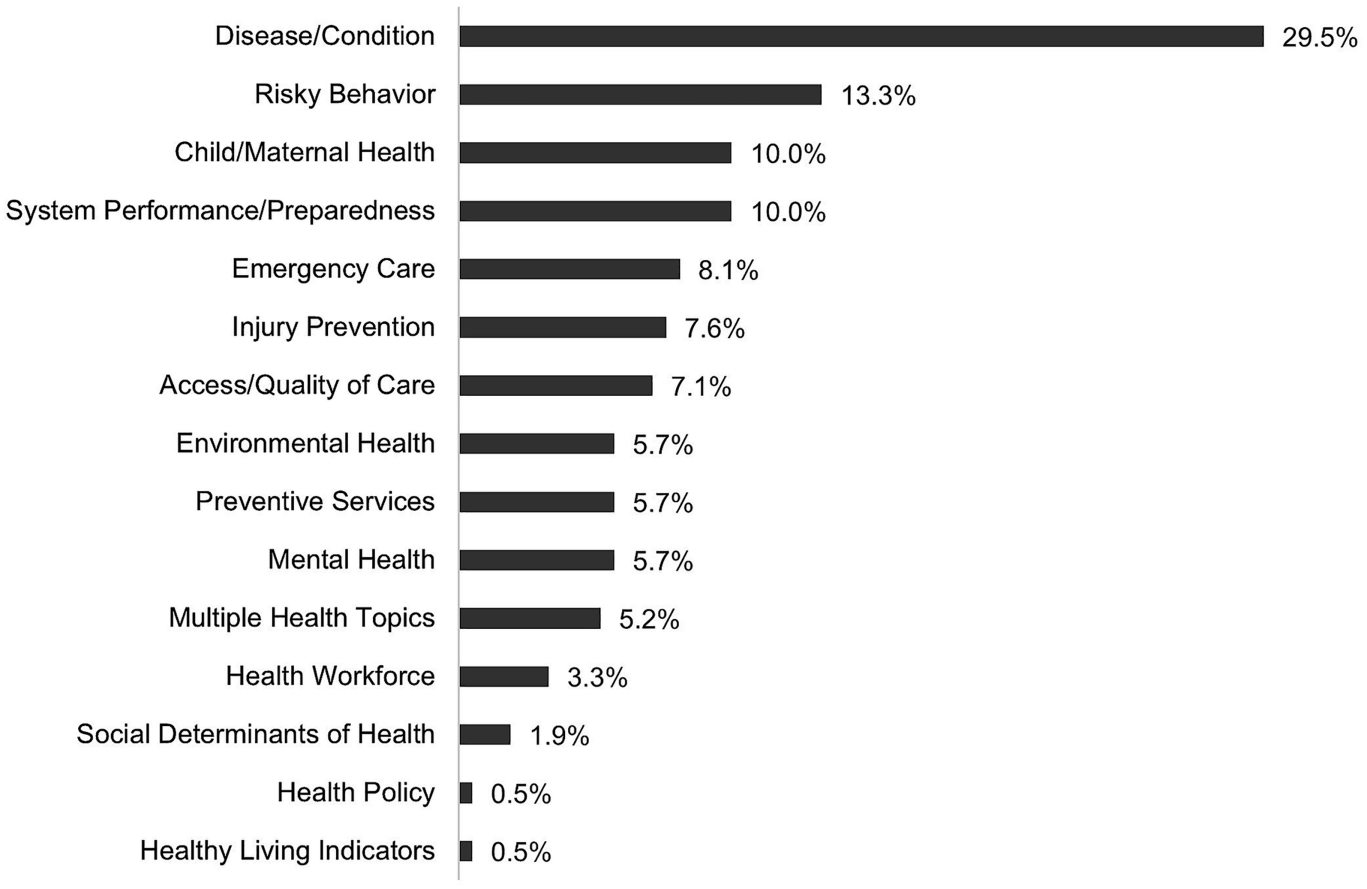
Distribution of health topics in U. S. federal and state public health data dashboards, 2024–2025 (*N* = 210).

Of the 210 dashboards sampled, 58 (28%) are federal and 152 (72%) are state dashboards. Eighty-five percent of all dashboards were created between 2017 and 2024 and 50% since 2022, demonstrating that use of public health dashboards is proliferating. The dashboards sampled are used for visualizing data on diverse health topics that we group according to major public health focus areas. As shown in Figure 1, about one-third of all dashboards sampled are disease or condition-specific (e.g., cancer, diabetes, influenza, etc.) and 13% are focused on risky health behaviors (e.g., drug use and tobacco consumption). Other than surveillance, dashboards are also commonly used for sharing health services data regarding child and maternal care, emergency care, injury prevention, preventive care, and indicators of access or quality of care. By comparison, a very small percentage of public health dashboards are designed to track trends in health policy, workforce development, and social determinants of health. Most dashboards (80%) do not include explicit information regarding the identity of the dashboard’s creator or source of funding, which has been repeatedly shown to undermine user trust in these tools (10).

Regarding access, most dashboards sampled (73%) are hosted on official federal or state health departments’ websites, however 36% of state dashboards are hosted on third-party platform (e.g., Tableau or Microsoft Power BI), which raises a potential concern regarding accessibility and sustainability of these dashboards if financial or other circumstances change. Indeed, 31 (20%) of state dashboards in the sample were temporarily inaccessible when attempted to be reaccessed in March or July 2025 either due to technical issues or because the original URL was changed (see Supplementary material). However, similar concerns can be raised regarding reliable access to federal government dashboards. When reaccessed in March 2025, 17 of 58 (or 30%) federally maintained dashboards were inaccessible (i.e., removed or archived to comply with President Trump’s executive orders) (31), specifically dashboards visualizing COVID-19, HIV/AIDS, and health inequities-related data. Access to eight (47%) of these dashboards has been restored by July 2025, although some were altered to restrict data analyses by sex and race/ethnicity and others now require users to register before gaining access to the dashboard.

### 3.1 Characteristics of data used in national and state public health dashboards

Table 1 compares the characteristics of data used in federal and state public health dashboards. Data collected by the federal government is used in over half of all dashboards sampled, including 98% of all national dashboards and 37% of all state dashboards. Most state dashboards (91%) use in addition or exclusively data collected by state health departments. Only a fraction of all dashboards (4%) incorporated local (i.e., county or municipal) health data, as such data may not be readily available. Publicly available data from other sources (e.g., health maintenance organizations and philanthropic foundations) is rarely used in either federal or state dashboards.

**Table 1:** Characteristics of data used in U. S. federal and state public health data dashboards, 2024–2025.

	All dashboards (*N* = 210)		Federal dashboards (*N* = 58)		State dashboards (*N* = 152)	
	*n*	%	*n*	%	*n*	%
Data source						
Federal agency*	114	54.3	57	98.3	57	37.5
State agency*	138	65.7	0	0	138	90.8
Local agency*	9	4.3	1	1.7	8	5.2
Research organization	7	3.3	1	1.7	6	3.9
Health care system	3	1.4	1	1.7	2	1.3
Foundation	1	0.5	0	0	1	0.7
Health care industry	1	0.5	0	0	1	0.7
International source	1	0.5	0	0	1	0.7
Data type						
Epidemiological*	93	44.3	17	29.3	76	50
Emergency care*	36	17.1	4	6.9	32	21.1
Health services*	69	32.9	15	25.4	54	35.5
Environmental hazard*	18	8.6	3	5.2	15	9.9
Socioeconomic	11	5.2	2	3.4	9	5.9
Behavioral*	26	12.4	4	6.9	22	14.5
Health outcomes*	24	11.4	5	8.6	19	12.5
Public opinion	2	1	1	1.7	1	0.7
Performance/preparedness*	26	12.4	12	20.7	14	9.2
Social determinants of health	6	2.9	2	3.4	4	2.6
Data granularity						
Local*	136	64.8	10	17.2	126	82.9
State*	182	86.7	37	63.8	145	95.4
Region	37	17.6	10	17.2	27	12.8
National*	65	31	58	100	7	4.6
International	2	1	2	3.4	0	0
Population						
General population*	113	53.8	24	41.4	89	58.6
Patient population	27	12.9	7	12.1	20	13.2
Provider population*	8	3.8	7	12.1	1	0.7
Health care organizations	9	4.3	4	6.9	5	3.3
Health services*	18	8.6	9	15.5	9	5.9
Health records or claims*	23	11	9	15.5	14	9.2
Health incidents or events*	16	7.6	3	5.2	13	8.6
Vulnerable population**	47	22.3	5	8.6	42	27.6

*p < 0.05, **p < 0.001.

Percentages for each variable add up to more than 100% because variables could be coded into multiple categories. The vulnerable population category refers to data specific to infants and children, older adults people, members of racial/ethnic minority groups, people experiencing poverty, people who live in rural areas, uninsured or underinsured individuals or households, people with undocumented status, individuals who identify as LGBTQ+, people with disabilities, people with mental illness, persons with substance use disorders, persons experiencing homelessness, and persons who are incarcerated.

Epidemiological and health services data are the most common types of data used in public health dashboards (44 and 33% of all dashboards, respectively) since both are routinely collected via existing data collection systems. Dashboards also commonly utilize risk behavior and health outcomes surveillance data (12.4 and 11.4% of all dashboards, respectively), with the latter primarily used in so-called “disparity dashboards” or dashboards that track indicators of health disparities (32). Federal dashboards are significantly more likely to use performance or preparedness-related data (21% compared to 9% of state dashboards) given stronger mandates concerning collection and reporting of such data at the federal level. In contrast, state dashboards are significantly more likely to utilize emergency care-related data (21% compared to 7% of federal dashboards), although this difference may be artificially inflated due to states’ increased use of dashboards to track opioid-related overdoses and emergency room visits to guide local response efforts. State dashboards are also more likely to include environmental risk data (35.5% compared to 25.4% of federal dashboards), which may reflect differences in priorities of federal and state governments. Lastly, very few public health dashboards (less than 4%) utilize public attitudes or social determinants of health data, despite their relevance to public health. This may be due to the cost and complexity of collecting such data, but also because it is not always clear how such data may be actionable from a public health perspective (33).

The findings in Table 1 also reveal important insights regarding representation of different populations in data used by federal and state dashboards. Federal and state dashboards routinely use general population and/or patient data. However, data collected from providers is more frequently used in federal dashboards compared to state dashboards (12% compared to 0.7% of dashboards, respectively), perhaps because states’ regulatory authority to collect and use provider data vary significantly. Federal dashboards are also slightly more likely than state dashboards to incorporate administrative and other data collected on healthcare organizations, health services, and health records or claims. Still, the most notable pattern of findings emerges regarding the representation of vulnerable populations. Data absenteeism, i.e., the chronic absence or limits of data collected on groups experiencing social vulnerabilities, is a major obstacle to both accurately documenting and addressing health inequities (34), and only 22% of all dashboards sampled utilized data specific to vulnerable populations. State dashboards were significantly more likely than federal dashboards to incorporate such data (27.6% vs. 8.6%, respectively), with about a third focusing exclusively on infant and child health indicators and 5% on data collected on Medicaid recipients. By comparison, dashboards that include data specific to other vulnerable groups, such as older adults people, people experiencing poverty and/or homelessness, persons who are incarcerated, and persons identifying as LGBTQ+, are scarce (less than 3% of dashboards).

### 3.2 Usability of national and state public health dashboards

Table 2 summarizes findings regarding the usability of public health data dashboards, specifically accessibility, ease of navigation, interactivity, data visualization, and availability of technical assistance. Only 22% of the dashboards sampled (55% of federal dashboards and 9% of state dashboards) claim to be ADA-compliant, raising concerns about the degree to which many existing dashboards are accessible to individuals with disabilities. Ease of navigation also improves when dashboards are accessible via a central (parent) webpage that allows users to access multiple dashboards on the same or different health topics (27). Our analysis reveals that most current dashboards (57%) are only accessible via a standalone webpage, although state dashboards are more likely to utilize multi-dashboard landing pages (47% compared to 31% of federal dashboards), potentially making them easier for users to access and navigate. This difference appears to be primarily due to federal dashboards being independently created by federal health agencies for topics that fall under their specific purview, which is not the case for dashboards designed and maintained by state health departments.

**Table 2:** Usability of U. S. federal and state public health data dashboards, 2024–2025.

	All dashboards (*N* = 210)		Federal dashboards (*N* = 58)		State dashboards (*N* = 152)	
	*n*	%	*n*	%	*n*	%
Accessibility and ease of navigation						
ADA-compliant website**	46	21.9	32	55.2	14	9.2
Dedicated (standalone) landing page**	120	57.1	40	69	80	52.6
Parent or main landing page**	88	41.9	18	31	70	46.1
Multi-dashboard hub**	130	61.9	27	46.5	103	67.7
Interactivity						
User-generated dashboard	2	1	0	0	2	1.3
Visualization download option	102	48.6	32	55.2	70	46.1
Data download option**	112	53.3	51	82.9	61	40.1
Data upload option	1	0.5	0	0	1	0.7
User feedback option**	66	31.4	25	43.1	41	27
Data visualization tools						
Maps**	157	74.8	34	58.6	123	80.9
Graphs	193	91.9	52	89.7	141	92.8
Tables	149	71	45	72.6	104	68.4
Timeline	1	0.5	0	0	1	0.7
Tooltip	154	73.3	41	70.7	113	74.3
Simulations	1	0.5	0	0	1	0.7
Technical support						
No information provided**	54	25.7	6	10.3	48	31.6
Use instruction on website**	150	71.4	50	86.2	100	65.8
Link to user manual**	19	9	13	22.4	6	3.9
Illustrations or examples of use**	34	16.2	20	34.5	14	9.2
Link to available training*	14	6.7	6	10.3	8	5.3
Contact info for inquires	67	31.9	17	29.3	50	32.9

*p < 0.05, **p < 0.001.

Percentages for each variable add up to more than 100% because variables could be coded into multiple categories.

Regarding interactivity, the findings in Table 2 suggest that federal dashboards are generally more interactive than state dashboards on most benchmarks assessed including options to download data, download a visualization, and provide user feedback. However, virtually none of the federal and state dashboards sampled offers users the option to generate visualizations tailored to their specific questions or information needs despite both options being feasible to implement using modern dashboard design technology.

Lastly, whereas virtually all federal and state dashboards appropriately utilize a range of data visualization tools (e.g., tables, graphs, and maps), they appear to be lacking in availability of technical support. Thus, one-quarter of the dashboards analyzed do not include any technical support information, and only about a third include contact information for inquiries. In addition, whereas most federal and state dashboards (86 and 66%, respectively) include use instructions on the website, only a handful, mostly federal dashboards, include links to user manuals, training resources, or examples of how the dashboard may be used.

### 3.3 Usefulness of national and state public health dashboards

Table 3 compares usefulness-related affordances of federal and state public health dashboards. Dashboards are presumed to be useful if they provide users with timely, relevant, credible and actionable information. As shown in Table 3, trust certificates are included in 97% of all federal dashboards compared to 42% of state dashboards, as dashboards created by federal agencies are required to carry an official trust certificate. Still, most federal and state dashboards (85%) do not explicitly identify the intended users of a dashboard or explain how and for what purpose they may use this tool, which may discourage the use of these tools by some.

**Table 3:** Usefulness of U. S. federal and state public health data dashboards, 2024–2025.

	All dashboards (*N* = 210)		Federal dashboards (*N* = 58)		State dashboards (*N* = 152)	
	*n*	%	*n*	%	*n*	%
Credibility						
Trust certificate included**	120	57.1	56	96.6	64	42.1
Intended users						
None specified	179	85.2	50	86.2	129	84.9
Researchers**	6	2.9	4	6.9	2	1.3
Health professionals**	12	5.7	2	3.4	10	6.6
Policymakers	4	1.9	1	1.7	3	2
General public*	9	4.3	4	6.9	5	3.3
All potential users*	12	5.7	2	3.4	10	6.6
Data affordances						
Epidemiological surveillance**	126	60.3	25	43.1	101	66.9
Risk surveillance**	35	16.7	5	8.6	30	19.9
Policy surveillance	1	0.5	0	0	1	0.7
Workforce surveillance*	6	2.9	4	6.9	2	1.3
System performance monitoring**	27	12.9	16	27.6	11	7.3
Capacity/gaps in access	23	11	7	12.1	16	10.6
Capacity/gaps in utilization**	31	14.8	4	6.9	27	17.9
Effects of social determinants	7	3.3	2	3.4	5	3.3
Projection of future trends	1	0.5	1	1.7	0	0
Customization affordances						
Indicators*	192	91.9	57	98.2	135	88.8
Demographics	115	55	30	51.7	85	55.9
Location	144	68.9	40	68.9	104	68.4
Time	135	64.6	36	62	99	65.1
Cases/conditions*	134	64.1	40	68.9	94	61.8
Organizations	24	11.5	6	10.3	18	11.8
Social determinants of health**	27	12.9	3	5.2	24	15.8
Query option*	26	12.4	5	8.6	21	13.8
Analytical affordance						
Descriptive analysis	210	100	58	100	152	100
Trend analysis	195	92.9	55	94.8	140	92.1
Comparison analysis	194	92.4	56	96.6	138	90.8
Multivariate analysis**	23	11	13	22.4	10	6.6
Choice analysis**	17	8.1	11	19	6	3.9
Predictive modeling or simulation	8	3.8	3	5.2	5	3.3
Program evaluation	3	1.4	0	0	3	2
Interpretation affordances						
Brief explanation/disclaimer	208	99	58	100	150	98.7
Visual techniques	122	58.1	34	58.6	88	57.9
Data storytelling*	6	2.9	0	0	6	3.9
Link to additional information**	158	75.2	54	93.1	104	68.4

*p < 0.05, **p < 0.001.

Percentages for each variable add up to more than 100% because variables could be coded into multiple categories.

Common data affordances of public health dashboard include epidemiological surveillance (60% of all dashboards), risk surveillance (16.7%), detection of gaps or disparities in utilization of health services such as screening and vaccination (14.8%), and monitoring performance of health systems on indicators such as hospitalizations (13%). State dashboards appear to be skewed toward using dashboards primarily for epidemiological and risk surveillance whereas federal dashboards use dashboards in addition to track and assess health systems performance. Other potential data affordances such as tracking health policy, social determinants of health data, and projecting trends are less common, although relevant pools of data are increasingly available (14). Customization affordances of dashboards follow a similar pattern: most federal and state dashboards allow users to compare distributions of variables or indicators across demographic groups (65% of all dashboards), geographical areas (78.5%), and time (75%), but significantly fewer offer users the option to disaggregate data by other relevant factors such as health insurance status (6.2%), risk profile (12.4%), or neighborhood characteristics (17%). Consequently, all dashboards analyzed can support descriptive data analyses, but only a handful can support more advanced data analyses, such as multivariate analysis (11%), choice analysis (8%), or predictive modeling (4%). This significantly limits the ability of users, particularly those with advanced data literacy and analysis skills, to analyze data in context or conduct causal analyses. Given this, it is reassuring that virtually all dashboards analyzed include a brief explanation or a disclaimer regarding data limitations and many (58%) use visual techniques to highlight major insights for users. Most (75%) also include links to external sources of additional information about relevant health topics or data for users who seek deeper insights, although use of data storytelling techniques remains scarce.

## 4 Discussion

Public health data dashboards have a significant potential to improve data access and use in health decision-making at the national, state, and local levels (5, 6, 8). Our findings demonstrate that dashboards are already being used extensively by federal and state public health agencies to equip health decision makers and the public with timely, relevant, credible, and useful data across a wide spectrum of public health issues and applications. However, our mapping and analysis of the current landscape of public health dashboards reveals that several critical challenges remain to realizing the full potential of these tools. A primary challenge is lack of a standard approach to design and implementation of usable and useful dashboards. Our findings point to significant differences in how, for whom, and for what purpose public health dashboards are created as well as in their relative ease of access and use to different users. Dashboards created by federal agencies are more likely to conform to uniform design standards than dashboards created by state public health departments, but the process of designing and implementing dashboards does not appear to follow systematic strategy focused on maximizing usability and usefulness. This likely reflects the current state of research and evidence to guide optimal design and implementation of dashboards, which remains mostly disjointed, lacking firm grounding in theories or frameworks that logically link usability and usefulness affordances to user experience and learning, and is limited to insights obtained from descriptive case studies as opposed to rigorous evaluations (13, 16–18).

A second challenge involves ensuring routine, reliable, and sustained access to these tools. We find that access to both federal and state public health data dashboards may be disrupted and become unreliable, but for different reasons: state dashboards rely heavily on third-party platforms for designing and hosting dashboards and are susceptible to disruptions caused by licensing or technical issues, whereas federal dashboards are susceptible to disruptions due to changes in federal policies and mandates regarding data collection and sharing. Public health data are critical public good, and federal government dashboards are especially susceptible to impacts of disappearing or altered public health data (35). Overall, robust access to data via dashboards is necessary to ensure these tools are usable, useful, and used consistently for supporting evidence-informed public health decisions at all levels.

Third, our findings highlight important gaps regarding the types, scope, and nature of data used in public health dashboards. First, data and indicators most frequently used in dashboards emphasize infectious disease, chronic conditions, mortality, and risk factor exposures, with less attention to upstream factors that impact population health such as social determinants of health, changing climate, and public health workforce development and training. Further, the use of unstructured data from alternative sources (e.g., social media or sensors) is scarce despite their potential to provide critical insights regarding lived experiences and flow of health information. Second, most public health data dashboards may be perpetuating chronic underrepresentation of members of socially vulnerable populations in public health data by not explicitly acknowledging inequitable representation and cautioning readers about limitations of inference from such data. Third, data collected by federal agencies are generally less granular than data collected by state health departments, which has implications for using national data for public health decision-making at the local level. Dashboards that pull and aggregate comparable data across state dashboards have the potential to overcome this issue.

Lastly, dashboards are most useful if they present data in context, yet most of the federal and state dashboards analyzed do not include features such as data storytelling or annotations that offer important insights regarding context, although there are positive signs of dashboard designers moving in this direction. We are not suggesting that such features ought or need to be incorporated into all dashboards—for example, context may matter more for strategic decisions than for operational ones—but rather propose that the potential value of including such features is considered in the design process.

### 4.1 Study limitations

This study utilized a cluster probability sampling of federal and state public health dashboards in the U. S. and therefore provides a more complete and nuanced representation of this landscape than that generated by similar studies that relied on purposive samples. In addition, this study employed a theory-grounded, valid, and reliable data extraction instrument with a particular focus on assessing usability and usefulness. The most notable limitations of this study are the exclusion of public health dashboards available from sources other than federal and state health agencies (e.g., foundations, health insurers, universities, and news media) and that usability and usefulness of dashboards were not directly assessed based on actual users’ feedback but rather assessed indirectly but examining usability and usefulness affordances. Despite these limitations, the study offers valuable insights regarding gaps and opportunities for realizing the full potential of data dashboards in improving and advancing evidence-informed public health decision-making.

### 4.2 Policy and practice implications

Federal and state agencies have a critical role in the collection, curation, analysis, and dissemination of data that public health stakeholders rely on for making decisions, and data dashboards are increasingly used for connecting users with such data. Realizing the full potential of dashboards to provide timely, relevant, credible, and actionable insights for informing sound policy and practice requires convergence on a common set of standards and best-practices at the federal and state levels for guiding design and implementation of usable and useful dashboards. It is critical that such standards and best-practices be evidence-based and emerge from collaborations between designers, users, and intermediaries, which calls for additional investments in theoretically grounded and methodologically rigorous research to clarify and advance the science underlying dashboard design. Additional investments are also needed to support all users of these tools by providing adequate training, technical assistance, and improved customer support. Beyond that, greater consideration ought to be paid in the design and implementation process to ensuring data equity, interoperability, transparency, and governance—all of which are critical for building trust in data and evidence—as well as to instituting quality control and performance benchmarks for guiding improvements.

## Data Availability

The raw data supporting the conclusions of this article will be made available by the authors upon request, without undue reservation.
