# Supplementary material for: Usability and usefulness of U. S. federal and state public health data dashboards: implications for improving data access and use
Source: Front Public Health. 2025 Nov 27;13:1699312. doi: 10.3389/fpubh.2025.1699312 (PMC12695813; doi:10.3389/fpubh.2025.1699312)
Supplement: Supplementary file 1 [file Data_Sheet_1.pdf]

PUBLIC HEALTH DATA DASHBOARDS CODING INSTRUMENT

INSTRUCTIONS:

Coding of variables should be based on explicitly stated information or features of data dashboards that are directly observables (e.g., navigation, visualizations, etc.). In general, the selection and application of applicable codes should not be based on assumptions or logical inference regarding usability and usefulness of a dashboard. Please be sure to flag any ambiguous coding decision you encounter for further team review using the comment box.

When applying an "Other" code, please include clarifying information. For example, if a dashboard represents data collected from or about a particular population that is not listed in the available response option, please enter a descriptor for this population (e.g., "newborns" or "military personnel").

**Information/Variable**	**Assigned Values/Codes**	**Specific Instructions**
DASHBOARD IDENTIFIERS		
Dashboard URL	Record the web address (URL) of the dashboard	
Dashboard name	Record full name and acronym, if applicable.	
Top-level domain	e.g., 'cdc.gov' or 'nibi.nih.gov'.	The top-level domain is intended to capture the identity of the agency, organization, or entity that created and/or hosts the dashboard.
Dashboard creator	1. None referenced<br>2. Referenced: _________	Look for this explicit information on the website (e.g., credits) or in any linked documentation. If identifying information is provided, please include it with your response.
Dashboard host	1. Federal government website<br>2. State government website<br>3. Third-party host (e.g., Tableau public)	Per top-level domain.
Year dashboard was created		If not indicated, use The Wayback Machine (https://web.archive.org/) to enter the web page's URL into the search bar and see when it first registered or crawled the page. This won't be an exact date, but it will provide a rough idea of when the web page was published.<br><br>Leave blank if no date can be determined.
Date dashboard was last updated or reviewed	Record date using the following format: dd/mm/yyyy	This information is typically reported at the bottom of the dashboard webpage.<br><br>Leave blank if no date can be determined.
DATA SOURCE AND CONTENT		
Data source(s)	1. Federal agency (e.g., CDC, AHRQ)<br>2. State agency (e.g., state health department)<br>3. Local agency (e.g., municipal, county)	Select all that apply.<br><br>*Note*: This item inquiries about the agency, organization, or entity that collected or provided the data used in the dashboard. This information

Information/Variable	Assigned Values/Codes	Specific Instructions
	4. Research organization (e.g., university, nonprofit research institute)<br>5. Healthcare system or organization (e.g., a hospital system)<br>6. Health insurance organization (e.g., Medicaid, Aetna, etc.)<br>7. Foundation (e.g., RWJF, KFF, or Urban Institute)<br>8. Public polling organization (e.g., Roper)<br>9. Healthcare industry or trade group (e.g., pharmaceutical or a professional association)<br>10. Media (news/social media, Internet searches)<br>11. Dashboard users<br>12. International sources (e.g., WHO)<br>13. N/A or unclear<br>14. Other ____	can typically be found in the technical notes provided or as a note attached to the visualization.<br><br>Be sensitive to cases where data are collected by a vendor such as Westat or RTI for an agency (in which case that agency is the source of data) as opposed to data collected and shared by the vendor (in which case the vendor is the data source).
Type of public health data	1. Epidemiological data (incidence of disease, morbidity/mortality, events such as drug overdoses)<br>2. Emergency care data (data related to emergency care treatment)<br>3. Health services data (data about services, e.g., hospitalization, ambulatory care, screens)<br>4. Environmental risk data (air/water quality, heat, ticks)<br>5. Social and economic data (health-related inequalities, financial cost)<br>6. Behavioral monitoring data (nutrition, physical activity, alcohol consumption, vaccination)<br>7. Health outcomes data (e.g., births, deaths, life expectancy, quality of life)<br>8. Health policy data (legislation, policies, regulations)<br>9. Public sentiment data (news coverage, social media posts, Google searches)<br>10. System performance or preparedness data (e.g., customer service, donor matching)<br>11. Social determinants of health data (e.g., social vulnerability index)<br>12. Other ___	Select all that apply.<br><br>*Note*: This item inquiries about the content of the data used in the dashboard. **Epidemiological data** are primarily about the frequency or distribution of diseases and conditions (e.g., number of COVID infections); **Emergency care data** refer to data on patient encounters, diagnoses, outcomes, and resource utilization within emergency departments (e.g., number of patients treated for COVID-related complications); **Health services data** refer to data collected within the healthcare system regarding services provided to patients (e.g., screening for birth defects); **Environmental risk data** refers to data about environmental hazards (e.g., air pollution); **Social and economic data** refer to data about actual or potential cost or burden of diseases and conditions (e.g., workdays lost due to influenza); **Behavioral monitoring data** are data obtained from self-reports about intentions or actions (e.g., percent of high school students reporting drug use); **Health outcomes data** refer to health and others outcomes experienced by individuals or groups (e.g., quality of life post-surgery); **Health policy data** refer to data on health-related legislation, rules, mandates, regulations, etc. (e.g., number of states mandating insurance coverage for mental health services); **public opinion/sentiment data** are data obtained from public opinion polls, news or social media, search engines data (e.g., opinions regarding vaccinations)**; System performance data** refer to data collected to assess a public health or a healthcare system performance or preparedness (e.g., response time to public inquiries regarding COVID isolation protocols); **Social determinants of health data** refer to data about the relationship between health and social determinants such as poverty (e.g., relationship between rates of people treated for lead poisoning and neighborhood characteristics).

Information/Variable	Assigned Values/Codes	Specific Instructions
Public health topic	1. Diseases and conditions (e.g., diabetes, cancer, HIV, flu)<br>2. Mental health (e.g., depression, anxiety)<br>3. Risky behaviors (alcohol, drug, and tobacco use; unsafe sex; seatbelt use, etc.)<br>4. Healthy living (healthy diet, exercise, elder care, etc.)<br>5. Preventive interventions or services (e.g., health screenings, vaccinations, preventive treatment)<br>6. Emergency care or medical treatment (e.g., surgeries)<br>7. Injury or violence prevention (e.g., gun violence or occupational safety)<br>8. Environmental health (e.g., air or water contamination, heat)<br>9. Public health workforce (e.g., doctors, nurses, etc.)<br>10. Health policy (laws, regulations, plans, etc.)<br>11. Preparedness or performance (e.g., of systems, agencies, infrastructure, etc.)<br>12. Social determinants of health (e.g., social vulnerability, economic disparities, etc.)<br>13. Other ____	Select all that apply.<br><br>*Note*: a selection should be made based on the title or the caption used to describe the dashboard and according to classes of general topics or major themes used by the CDC (see health topics webpage) and Healthy People 2030 (see https://odphp.health.gov/healthypeople/objectives-and-data/browse-objectives). Use the 'other' option for additional topics not listed or request a team review if you are unsure about the correct code to apply.
Data granularity	1. Local (city, town, county)<br>2. State<br>3. Regional<br>4. National<br>5. International<br>6. Other ______	Select all that apply.<br><br>*Note*: this item asks about the granularity of the actual data (property of the data), not how data may be broken down by level (a property of dashboard design). This information may be provided in the technical notes or description of the data collection methodology.
Populations represented in the data	1. General population (e.g., all adults nationally)<br>2. Patient population (regardless of disease or illness)<br>3. Provider population (doctors, nurses, etc.)<br>4. Organizations (e.g., clinics, public health departments, service providers, treatment facilities, etc.)<br>5. Services (e.g., hospitalizations, vaccination)<br>6. Laws, rules, regulations, etc.<br>7. Artifacts (e.g., administrative records, insurance claims)<br>8. Incidents or events (e.g., drug overdose, ER visits, etc.)<br>9. Other ___	Select all that apply.<br><br>*Note*: this item refers to the collection of units or cases (humans and otherwise) represented in the data.
Vulnerable subpopulations represented in the data	1. None<br>2. Infants and children<br>3. Older adults<br>4. People from racial/ethnic minority groups<br>5. People with lower socioeconomic status	Select all that apply.<br><br>*Note*: this variable applies only if data were exclusively collected from members of one or more vulnerable groups OR if a procedure (e.g.,

Information/Variable	Assigned Values/Codes	Specific Instructions
	6. People who are uninsured<br>7. People self-identified as LGBTQ<br>8. Immigrants or people with undocumented status<br>9. People who live in rural areas<br>10. People with disability<br>11. People with a mental illness<br>12. People experiencing homelessness<br>13. People who are incarcerated<br>14. People who are pregnant<br>15. Other ___	oversampling) was implemented to ensure adequate representation.<br><br>The categories listed are adopted from the CDC (https://www.cdc.gov/environmental-health-tracking/php/data-research/populations-vulnerabilities.html) and are meant to be mutually exclusive. If a subpopulation is defined based on the intersection of two or more vulnerability criteria (e.g., immigrant children or people with mental illness who are incarcerated), please record this information under the "Other" category as opposed to selecting multiple categories.
DASHBOARD USABILITY*		
ADA-compliant website?	1. Yes<br>2. No	Look for any explicit indication or statement that the website is ADA-compliant or a link to an accessible (e.g., text only) version of the dashboard.
Trust certification	1. Yes<br>2. No	Look for any explicit indication that this is an official federal or state government dashboard or a seal (see for example bottom of this page https://www.immport.org/shared/home).
Dashboard accessibility	1. A dedicated (standalone) landing page<br>2. Parent or main landing page with a directory of dashboards<br>3. User generated from choice of topic or indicators<br>4. Other ______	This question inquiries about how users navigate to the dashboard.<br><br>A standalone landing page refers to cases where a dashboard is accessed via a unique, unlinked URL, such that the URL takes users directly to the visualization, several visualizations on the same page, or a page that combines text with an embedded dashboard (see for example, https://www.cdc.gov/nchs/nvss/vsrr/mortality-dashboard.htm).<br><br>In some cases, users may access a dashboard via a directory of dashboards or a parent landing page that includes links to topic-relevant dashboards or other resources (For example, this dashboard https://www.cdc.gov/ticks/data-research/facts-stats/tick-bite-data-tracker.html is accessible from this parent page: https://www.cdc.gov/ticks/data-research/facts-stats/). To check for a potential parent page, shorten the dashboard URL to the nearest subdirectory shown in the address (e.g., https://www.cdc.gov/ticks/data-research/facts-stats/ in this case).<br><br>Another way users may access a visualization of interest is via dashboards that allow them to generate a visualization based on choice of topic and indicators, see for example, https://pegsexplorer.niehs.nih.gov/).
Multi-dashboard application?	1. Yes<br>2. No<br>3. Unsure (team review is needed)	A multi-dashboard application is a dashboard application that allows users to access different data visualizations from the same window via tiles

Information/Variable	Assigned Values/Codes	Specific Instructions
		or navigation menu<br>(e.g., https://wwwn.cdc.gov/norsdashboard/).
Data visualization tools	1. Maps<br>2. Graphs / charts<br>3. Tables<br>4. Timeline<br>5. Text (word cloud)<br>6. Tooltip or hovering<br>7. Animations/simulations<br>8. Other ___	Select all that apply.<br><br>*Note*: graphic representations of trends should be coded as graphs/charts. A timeline describes data in relation to events or milestones (e.g., timeline of the COVID-19 pandemic). Animations are dynamic presentations of data (including simulations).
User instructions and technical assistance	1. No instructions are provided.<br>2. User instructions are provided (on website or link to documentation)<br>3. Dashboard-specific technical information is included in FAQ<br>4. Examples or illustrations of dashboard use are provided<br>5. Links to training sessions or videos (e.g., webinars)<br>6. Dedicated contact info for responding to inquiries or questions (e.g., email)<br>7. Other ______	Select all that apply.
Data download option?	1. Yes<br>2. No	
Visualization download option?	1. Yes<br>2. No	
Data upload option?	1. Yes<br>2. No	
User Feedback option	1. Yes<br>2. No	
DASHBOARD USEFULNESS**		
Intended users	1. None specified<br>2. Scientists/researchers<br>3. Public health professionals/practitioners<br>4. Policymakers (legislators, regulators, decision-makers, etc.)<br>5. General public<br>6. Other ____	Select all that apply.<br><br>*Note*: look for this explicit information on the website (e.g., "about" or FAQ) or in any linked documentation; do not speculate about potential audiences.
Data affordances (goal or purpose of presenting data)	1. Epidemiological surveillance (incidence of disease, illness, or conditions)<br>2. Risk surveillance (monitoring existing or potential health risks and hazards, e.g., risky behaviors, outbreaks, climate-caused emergencies, etc.)<br>3. Policy surveillance (tracking legislation, regulations, etc. that can impact health)<br>4. Workforce surveillance (tracking changes or trends in current and future public health workforce)	Select all that apply.<br><br>*Note*: this variable is intended to capture the way the dashboard is intended (or expected) to be used such that it facilitates users' awareness, knowledge, and understanding of an issue; ability to interpret, analyze, and evaluate relevant evidence presented to draw conclusions; make specific decisions and develop a plan of action; and/or engage stakeholders.

Information/Variable	Assigned Values/Codes	Specific Instructions
	5. System performance or preparedness evaluation (assessing system capacity or readiness to meet current and future needs and challenges)<br>6. Assessing gaps or disparities in access to health care services or resources.<br>7. Assessing gaps or disparities in utilization of health care services or resources.<br>8. Exploring effects of social determinants of health and causes of health disparities<br>9. Projections of future trends or health outcomes<br>10. Evaluation of intervention or policy effects<br>11. Other ___	Ideally, the coding of this variable is based on the information provided on the website (e.g., "about" or FAQ) or in any linked documentation; However, if such information is not available, you may infer purpose from the type of data included in the dashboard and include a request for team review.
Predictive modeling or simulations (e.g., future trends)	1. Included<br>2. Not included	Predictive modeling may include projections (of future trends) or simulations (experiment with outcomes of different scenarios)
Data customization affordances	1. Selecting/filtering indicators<br>2. Sorting/grouping (demographics)<br>3. Sorting/grouping (location)<br>4. Sorting/grouping (time)<br>5. Sorting/grouping (cases)<br>6. Sorting/grouping (organizations)<br>7. Sorting/grouping (social determinants of health)<br>8. Searching / query support<br>9. Other ___	Select all that apply.<br><br>*Note*: data customization refers to dashboard affordances regarding presentation of data; it is not necessarily the same as data disaggregation options (which has to do with the information recorded in the data)
Analytical affordances (types of analyses that can be performed using the dashboard)	1. Descriptive analysis (frequency counts, distribution)<br>2. Trend or historical analysis<br>3. Comparison of groups, cases, or outcomes<br>4. Multivariate analysis (probing interrelationships or associations among several variables or indicators, e.g., by applying layers).<br>5. Choice analysis (direct comparison of two or several alternatives, e.g., cost of fee-for-service vs. value-based care)<br>6. Predictive analysis (prediction or estimates of future trajectories or events).<br>7. Evaluation of interventions or policies<br>8. Other ____	Select all that apply.
Data interpretation and/or contextualization affordances	1. Brief explanation or disclaimer<br>2. Visual techniques (e.g., use of icons or colors to draw attention to key findings)<br>3. Data storytelling feature. If so, please include a brief description in the comments section.<br>4. Links to additional information or external resources<br>5. Other ___	Data storytelling communicates insights from data using a combination of narrative and visuals (such as infographics and articles) to aid interpretation. Please concisely describe data storytelling techniques or elements observed.<br><br>Select all that apply.

Notes:

* Usability refers to users' perception of how consistent, efficient, productive, organized, easy to use, intuitive, and straightforward it is to accomplish tasks within a system. Morales-Vargas et al (2023)[1] identify three approaches to usability evaluation: (1) functional (assessing a website's inherent characteristics, including its content, information architecture and visual design, as well as its technical and operational features, linked to technology and security); (2) experiential (focuses on user experience and perceptions and examines such factors as usability, accessibility, satisfaction and interaction); and (3) strategic (assessing websites in relation to site owner's objectives in terms of performance, visibility and positioning). Of the three, the functional approach is most suitable for evaluation via coding and analysis of data dashboards without engaging users directly, but it requires expertise in dashboard design which may diverge from users' assessment (which seems more relevant to assessing actionability). For this reason, the indicators of usability included in this coding scheme are limited to directly observable features or affordances of dashboards.

** Usefulness or utility refers to users' assessment of value, i.e., the degree to which the insights obtained from a dashboard are relevant, timely, credible, and responsive to users' needs and/or integrated with their workflow and decision-making routines. A direct measure of usefulness therefore requires gauging users' own assessment. An indirect approach to assessing usefulness is to code for potential data exploration and analysis affordances of using a dashboard that can facilitate users' learning, comprehension, interpretation, and generation of data-based insights, frequently referred to as actionability.[2]

---

[1] Morales-Vargas, A., Pedraza-Jimenez, R., & Codina, L. (2023). Website quality evaluation: a model for developing comprehensive assessment instruments based on key quality factors. *Journal of Documentation*, *79*(7), 95-114.

[2] Verhulsdonck, G., & Shah, V. (2022). Making actionable metrics "actionable": the role of affordances and behavioral design in data dashboards. *Journal of Business and Technical Communication*, *36*(1), 114-119.
